# Effects of Collective Histone State Dynamics on Epigenetic Landscape and Kinetics of Cell Reprogramming

**DOI:** 10.1038/srep16746

**Published:** 2015-11-19

**Authors:** S. S. Ashwin, Masaki Sasai

**Affiliations:** 1 Department of Computational Science and Engineering, Nagoya University, Nagoya, 464-8603, Japan

## Abstract

Cell reprogramming is a process of transitions from differentiated to pluripotent cell states via transient intermediate states. Within the epigenetic landscape framework, such a process is regarded as a sequence of transitions among basins on the landscape; therefore, theoretical construction of a model landscape which exhibits experimentally consistent dynamics can provide clues to understanding epigenetic mechanism of reprogramming. We propose a minimal gene-network model of the landscape, in which each gene is regulated by an integrated mechanism of transcription-factor binding/unbinding and the collective chemical modification of histones. We show that the slow collective variation of many histones around each gene locus alters topology of the landscape and significantly affects transition dynamics between basins. Differentiation and reprogramming follow different transition pathways on the calculated landscape, which should be verified experimentally via single-cell pursuit of the reprogramming process. Effects of modulation in collective histone state kinetics on transition dynamics and pathway are examined in search for an efficient protocol of reprogramming.

Differentiated mouse cells can be reprogrammed to induced pluripotent stem cells (iPSC) by inducing certain proteins known as Yamanaka factors (Oct4, Sox2, Klf4, and c-Myc) in the cell^1^. Though the precise mechanism of how Yamanaka factors (YF) reprogram remains elusive, clues to determining the mechanism should be obtainable from reprogramming pathways^2^,^3^,^4^. On inducing YF, marker genes of the differentiated cells are silenced in the early phase, and pluripotency genes such as *Nanog* become active only in the late phase, showing that the observed pathway of reprogramming is different from the pathway of differentiation. Therefore, theoretical analysis of how pathways are determined by gene regulation has been a focus of recent interest^5^,^6^,^7^,^8^,^9^.

Our understanding of gene regulation in differentiation and reprogramming has been advanced particularly by using the concept of epigenetic landscape^10^,^11^,^12^,^13^,^14^,^15^. In the landscape picture, stable cell states are represented by basins on the landscape while transition pathways between cell states are determined by topological connectivity among basins. Epigenetic landscape has been calculated in a variety of scenarios^6^,^9^,^16^,^17^,^18^,^19^,^20^,^21^, which has shown that landscapes have multiple basins corresponding to differentiated and embryonic stem cell (ESC) or iPSC-like pluripotent states; in order to understand transition pathways, it is necessary to elucidate the distribution of basins and connectivity among them on the epigenetic landscape.

Analysis of the structure of epigenetic landscape so far has been based on the assumption that gene activity is determined by binding/unbinding of transcription factors (TF) as in the case of bacterial gene regulation. However, in differentiation and reprogramming, genes are regulated not only by TF binding/unbinding but also by epigenetic state change including DNA methylation/demethylation, chemical modifications of histones, and the associated change in chromatin structure^22^,^23^. Therefore, in order to understand epigenetic landscape quantitatively, we need to develop a theoretical framework that explicitly takes epigenetic dynamics into account^16^,^24^. It should be noted that because of single-molecule nature of DNA, access of regulatory proteins to the gene loci is noisy, leading bursty transcription^25^. Therefore, in order to incorporate noisy genetic and epigenetic influences on the epigenetic landscape, we develop a theoretical framework based on the master equation^16^.

The epigenetic modification of a nucleosome is known to cause recruitment of modifier enzymes effecting the neighboring nucleosomes and causing them to behave similarly^26^. Theoretical^27^,^28^,^29^,^30^,^31^,^32^ and experimental^29^,^30^ studies have shown that this non-local interaction should bring about the collective change of many nucleosomes in a gene locus to show the discrete switching behavior. In particular, two insightful models^27^,^32^ have been proposed on how memory arises at the epigenetic level by taking long-range interactions of nucleosomes into account^27^ or by modeling short-range interactions with a Potts-like model^32^. Based on these observations, we refer to the collective histone state around a gene locus, which constitutes tens or more of similarly modified histones, as the *collective histone state* (CHS). In Fig. 1 we describe the coarse graining of many-histone states to CHS as a three state switch (*s* = 1,0,−1): The state (i) *s* = 1 corresponds to the loosely packed chromatin state with histones being actively marked and the gene can express itself, (ii) *s* = −1 is the tightly packed state with histones being repressively marked and the gene being silenced, and (iii) in the *s* = 0 state chromatin fluctuates between tightly and loosely packed structures with histones bearing neither repressive nor activating marks as in some loci of ESC^33^. We assume that binding of activator to gene regulatory region of DNA enhances the probability of the *s* = 1 state and binding of repressor enhances the probability of the *s* = −1 state, so that the gene activity, *i.e.* rate of protein synthesis in the model, is regulated by TF binding/unbinding through the CHS change.

In the present paper, we focus on the role of timescale difference among different processes. Effects of the timescale or the frequency of DNA state change on gene expression have been intensively studied by using the ratio of the rate of DNA state change to the rate of protein-copy-number change as a measure^11^,^19^,^34^,^35^,^36^,^37^,^38^,^39^,^40^,^41^. This measure is called *adiabaticity*, and the dynamics is *adiabatic* when this ratio is large and *non-adiabatic* when it is small. In the present case, timescale of DNA state change should be determined by epigenetic CHS dynamics; therefore, adiabaticity is measured by 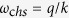, where *q* is the typical rate of change in CHS and *k* is the rate of protein degradation with the system being adiabatic when 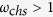 and non-adiabatic when 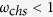. We show that adiabaticity of epigenetic state dynamics at each gene locus considerably affects the topological structure of the landscape and the slow non-adiabatic epigenetic CHS dynamics brings about the difference in pathway between differentiation and reprogramming in the model. Using this network model, we calculate the kinetic process of cell state transition and compare it with the observed data of reprogramming^42^,^43^,^44^. The simulated kinetic behavior predicts a characteristic histone-state change along the pathway, which should be examined by single-cell pursuit of the reprogramming process.

## A network model

**Figure 2:**
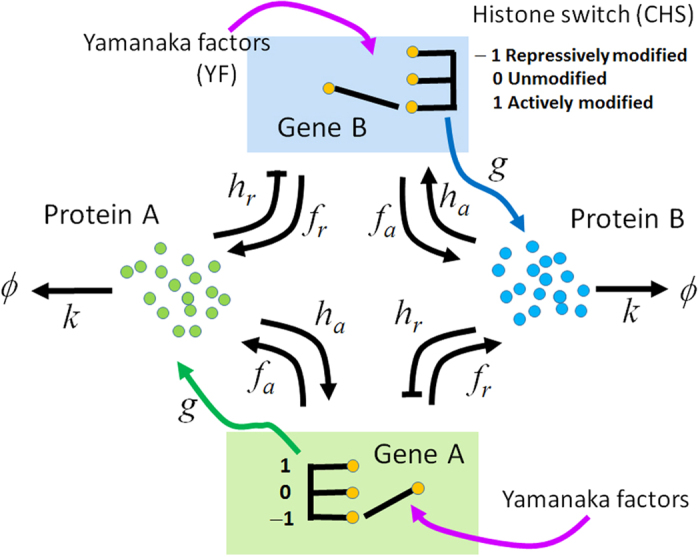
A schematic of the MRSA gene regulatory network in connection with the three state epigenetic switch. YF bind as pioneer factors^56^,^57^ to change the CHS. The 3-state CHS switch controls the protein production rate *g* as shown.

As a minimal model of cell-state transition, we consider the two gene mutual-repressor-self-activator (MRSA) regulatory model. As shown in Fig. 2, proteins produced by genes **A** and **B** repress each other, but positively regulate their own expression. Let *N*_*A*_ and *N*_*B*_ be copy numbers of proteins synthesized from **A** and **B**. Because **A** and **B** work in an antagonistic way, the model shows switching transition between the 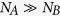 and 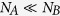 states. This **A** - **B** motif is ubiquitous in regulating differentiation as *Cdx2*- *Oct4* and *Gata6*- *Nanog*, for example^45^,^46^,^47^. As in these example motifs, we regard **A** as a marker gene specific to a differentiated cell and **B** as a pluripotency gene such as *Nanog*. We then study reprogramming as a transition from the differentiated cell with 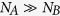 to the iPSC state with 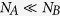. Each gene in the MRSA network is regulated through CHS by assuming that the rate of protein synthesis from the gene is large only when *s* = 1 and rates with which *s* changes depend on the activator or repressor binding status. It should be noted that the MRSA model without the inclusion of CHS dynamics has earlier been used as a prototypical model of differentiation^12^,^13^,^18^,^19^,^20^.

Along with *N*_*A,B*_, each gene status is described as  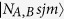  with CHS (*s* = −1,0,1), the repressor-binding state (*j* = 0: binding, *j* = 1: unbinding), and the activator-binding state (*m* = 1: binding, *m* = 0: unbinding). Protein-production rate, *g*_*sjm*_, is maximal for *g*_111_ = *g*, and we choose *g*/*k* = 1000 to fit a typical protein-copy number of eukaryotic TF^48^. Other *g*_*sjm*_ are chosen as *g*_110_ = 0.8 *g*, *g*_101_ = 0.1 *g*, and *g*_100_ = 0. For the CHS *s* = 0 and −1, we use *g*_011_ = 0.2 *g* with all other *g*_0*jm*_ = 0 and *g*_−1,*jm*_ = 0. Protein degradation rate is *k* ≈ 0.1 h^−1^ 49, and length of a cell cycle is about 2*k*^−1^ 42 though we do not consider cell cycle explicitly. For simplicity, we adopt same values of *k* and *g*_*sjm*_ for A and B. We consider that the rate constant of protein binding *h* is affected by other factors not involved in the present network model, which competitively or cooperatively interact with A and B when they bind to DNA; therefore, we assume the binding rate constants *h*_*a*_(*A*), *h*_*a*_(*B*) for activators and *h*_*r*_(*A*), *h*_*r*_(*B*) for repressors take different values. We assume proteins bind to DNA as a dimer^9^, so that the rates of binding are *h*_*a*_(*α*)*N*_*α*_(*N*_*α*_−1) and *h*_*r*_(*α*)*N*_*β*_(*N*_*β*_−1) with *α*,*β* = A, B, or B, A.

iPSC are unstable when they are cultured in medium without Lif, and stabilized when they are cultured in ES medium which contains Lif. Therefore, the iPSC state with 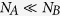 should have a shallow basin when Lif is withdrawn from medium and a relatively deeper basin when cells are cultured with ES medium. We represent this difference in stability of the iPSC state by assuming **A** and **B** are *asymmetric* in the case of unstable pluripotency. We model the asymmetry using *h*_*r*_(*B*) = 10 *h*, *h*_*r*_(*A*) = *h*, *h*_*a*_(*A*) = 4 *h*, and *h*_*a*_(*B*) = *h/4*. In the case showing the stable iPSC state, we assume, for simplicity, **A** and **B** are *symmetric* with 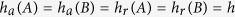. Rates of unbinding of TF from DNA are denoted by 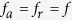. We set *f*/*h* = 50000 to make the ratio 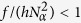. Following the observed data of single-molecule measurement^50^, we assume that TF binding/unbinding is fast enough as *f*/*k* = 10; With such fast TF binding/unbinding, the other slower process, the CHS transition, should be a key determinant of adiabaticity of the DNA state change in the present model.

Rates of stochastic CHS transitions are *q*_*jm*_ for *s* = 0→1, *r*_*jm*_ for *s* = 0→1, 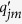 for *s* = −1→0, and 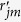 for *s* = 0→−1, where 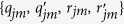 are chosen as real multiples of a tuning rate *q*. Therefore, the adiabaticity measure is 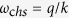. We assume that the CHS tends to be turned active when the activator binds, and turned repressive when the repressor binds to DNA. We therefore, have 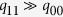 and 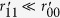. See Methods for further details.

## Results

We first study how topology of epigenetic landscape is influenced by the CHS dynamics, and then discuss pathways and kinetics on the landscape.

### Topology of epigenetic landscape

**Figure 3:**
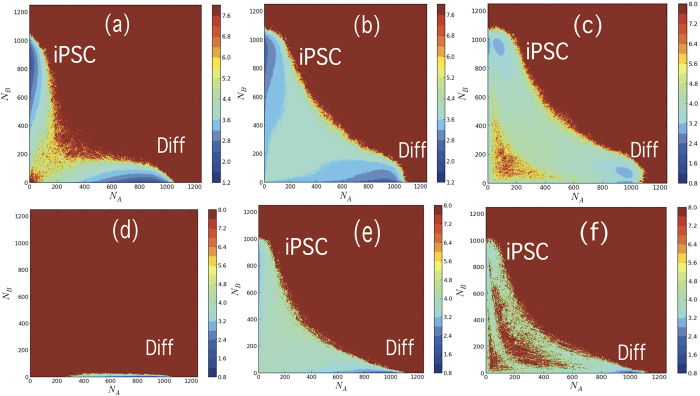
Epigenetic landscape *U(N*_*A*_*, N*_*B*_) when CHS dynamics is (a,d) adabatic: 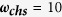, (b,e) intermediate: 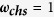, and (c,f) non-adiabatic: 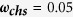
. Symmetric (**a–c**) and asymmetric (**d–f**) networks.

We calculated steady state distribution *P*_*s*_(*N*_*A*_, *N*_*B*_) by simulating the stochastic equations described in Methods using the Gillespie algorithm^51^ to derive the epigenetic landscape: 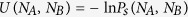. 100 trajectories were used for sampling, each over 10^8^ Gillespie-steps long with random initial conditions. The role of CHS switching is studied in the spectrum of adiabatic to non-adiabatic timescales. Figure 3 shows topological changes manifesting on the epigenetic landscape in both symmetric and asymmetric models.

In symmetric landscapes, two distinct states appear as two basins. In the strongly adiabatic case with 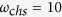 (Fig. 3a), features of CHS dynamics are averaged out, and two basins are separated by a large epigenetic barrier as in the MRSA network without CHS dynamics. Here, a path connecting two basins through the barrier is referred to as *diagonal pathway*. With the intermediate adiabaticity of 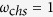 (Fig. 3b), the barrier is washed away due to the resonance between CHS dynamics and protein copy-number dynamics. This flat landscape should result in the widely fluctuating cell states, which disagree with the observed narrow distribution of cell states along the reprogramming pathway. In the non-adiabatic case with 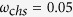 (Fig. 3c), both differentiated and iPSC states are stably formed, and we find the emergence of a stable intermediate state with 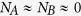. This novel intermediate state is connected to the differentiated as well as the iPSC states through low barrier pathways (valleys). Here, we refer to these valleys arising out of non-adiabatic CHS dynamics as *epigenetic valleys* or *epigenetic pathway*.

In asymmetric landscapes, the differentiated state has a deeper basin due to the enhanced stability. In adiabatic case with 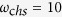 (Fig. 3d), the iPSC basin vanishes. In the intermediate case with 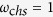 (Fig. 3e), the population widely spreads to give a flat landscape as in the symmetric model, which does not support a stable cell state. In the asymmetric non-adiabatic case with 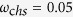 (Fig. 3f), we find differentiated, iPSC, and intermediate basins, which are connected by diagonal and epigenetic pathways, and further, we find another low-lying pathway in between diagonal and epigenetic pathways, which is characteristic to asymmetric non-adiabatic landscape.

We have shown that the topology of the epigenetic landscape is decisively dependent on the adibaticity of the CHS dynamics. We should note that without the CHS dynamics, the landscape does not have epigenetic valleys or the intermediate state with 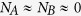, but only shows a diagonal pathway as in Fig. 3a. In the next subsection we examine the epigenetic pathway induced by the non-adiabatic CHS dynamics and its role in the reprogramming mechanism.

### Pathways of transitions between cell states

We investigate the role of pathways induced by the CHS dynamics by numerically solving the master equation of the model. See Methods for the explicit form of the equation and the calculation details.

Since ES medium is used in reprogramming, we use the symmetric network model to study it. The precise mechanism of YF action is not known; therefore, we compare two possible mechanisms; (I) YF work as histone-mark erasers by changing the CHS as 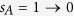 and 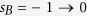, and (II) they work as activators on **B** as 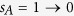 and 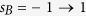. We simulate reprogramming by using a relative importance factor 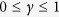; *γ* = 1 when YF solely act as histone-mark erasers, and *γ* = 0 when they are efficient to activate the CHS in **B**. Thus, the action of YF is represented by a matrix in the master equation, 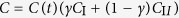, where **C**_I_ and **C**_II_ represent the above mechanisms I and II (see Methods), and 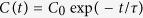 is the effectiveness of YF with *τ* being the lifetime of ectopic expression.

**Figure 4:**
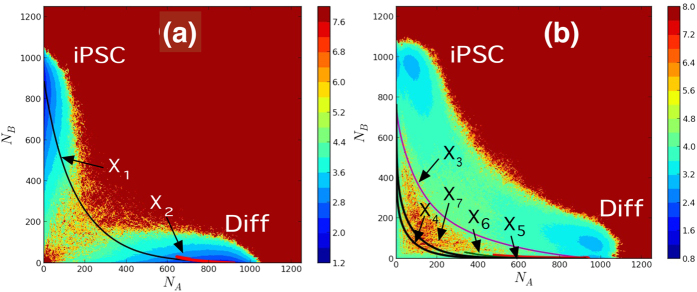
Reprogramming trajectories, *X*(*t*), are drawn on the epigenetic landscape, *U*(*N*_*A*_, *N*_*B*_). (**a**) Adiabatic (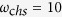) and (**b**) non-adiabatic (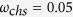) cases. See text for details.

Various processes of reprogramming are compared in Fig.   4 by plotting trajectories 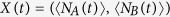 on the landscape, where 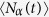 for *α* = A and B is the average, 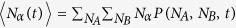. Reprogrammed trajectories start from the equilibrium differentiated state. In the adiabatic case with 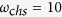, we show two trajectories *X*_1_(*t*) and *X*_2_(*t*) with efficiency *C*_0_/*k* = 100 and 10, respectively (Fig. 4a). The adiabatic nature of the CHS dynamics makes the trajectories independent of the protocol *γ*. YF is inefficient in the case of *X*_2_(*t*) causing a reversal, in contrast *X*_1_(*t*) is able to reach the iPSC state, this signifies that 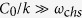 for YF to be sufficiently effective. In the non-adiabatic case with 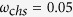 (Fig. 4b), we study five trajectories *X*_3_(*t*) with *γ* = 0 and *C*_0_/*k* = 0.1 and *X*_4_(*t*), *X*_5_(*t*), *X*_6_(*t*), *X*_7_(*t*) with *γ* = 1 having efficiency *C*_0_/*k* = 5.0, 0.05, 0.1, 0.5, respectively. Starting from the differentiated state, owing to a largest efficiency, *X*_4_(*t*) gets closest to the intermediate state and surpasses the epigenetic barrier to reach the iPSC. *X*_5_(*t*) and *X*_6_(*t*) have small efficiencies, so that they reverse back to the differentiated state. *X*_7_(*t*) with a medium efficiency crosses the epigenetic barrier but does not get as close to the intermediate state as *X*_4_(*t*). On keeping *γ* = 1 fixed and increasing *C*_0_, the system goes closer to the intermediate state. By decreasing *γ* and keeping *C*_0_ fixed, on the other hand, the trajectories depart from epigenetic valleys. as is apparent with the case *X*_3_(*t*). Trajectory *X*_3_(*t*) bypasses the epigenetic barrier suggesting that rapid reprogramming is realized along this pathway. Thus, the pathway of reprogramming sensitively depends on the way how YF work when the CHS dynamics is non-adiabatic. We can expect this rich behavior when kinetics of reprogramming is experimentally analyzed.

**Figure 5:**
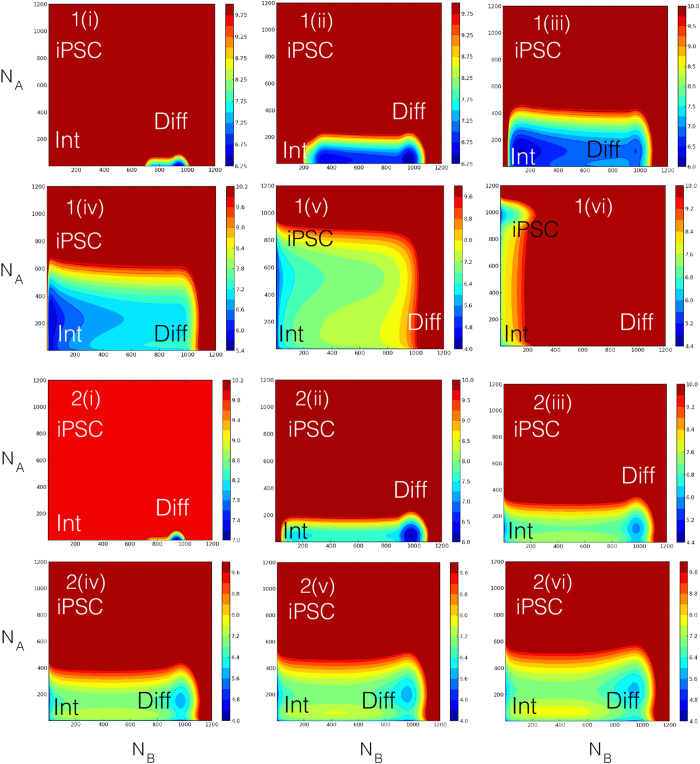
Evolution of the probability distribution *P*(*N*_*A*_*,N*_*B*_*,t*) from the differentiated to iPSC basins. Symmetric model was simulated under the non-adiabatic condition (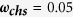). *P*(*N*_*A*_,*N*_*B*_,*t*) are shown for **(1)** *X*_4_(*t*) with *C*_0_ = 5.0*k* and *γ* = 1 at time instances (i) *t* = 0.3/*k*, (ii) 1.5/*k*, (iii) 3.6/*k*, (iv) 4.5/*k*, (v) 7.5/*k*, and (vi) 15/*k*, and **(2)** *X*_6_(*t*) with *C*_0_ = 0.1*k* and *γ* = 1 at time instances (i) *t* = 0.3*k*, (ii) 3.0/*k*, (iii) 9/*k*, (iv) 18/*k*, (v) 36/*k*, and (vi) 90/*k*. At *t* = 0, the entire distribution is confined to the differentiated basin. Note how the distribution evolves towards the intermediate state.

In Fig. 5, we show the time evolution of the probability distribution, 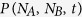, for the *X*_4_(*t*) (1(i)–(vi)) and *X*_6_(*t*) (2(i)–(vi)) cases with 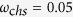. Here, 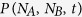 starts at time *t* = 0 from the population confined in the differentiated basin. This result shows that with the non-adiabatic CHS dynamics, reprogramming proceeds through epigenetic valleys, which are absent both in models without CHS dynamics and the adiabatic model. Starting from the differentiated state basin, the population approaches the intermediate state at 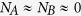. The approach to the intermediate state is very clear in *X*_4_(*t*), where the significant part of the population enters the intermediate state. In the case of *X*_6_(*t*) which exhibits reversal, the major part of 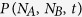 remains near the differentiated state, but the minor part proceeds along the epigenetic valley and reaches the intermediate state. Importantly, this approach to the intermediate state in the non-adiabatic reprogramming process is consistent with the experimentally observed late activation of the pluripotency genes after the lineage specific genes being repressed^2^,^3^,^4^. Since only the non-adiabatic case explains the pathway through such intermediate state, and epigenetic valleys are absent with the fast adiabatic CHS dynamics, the model strongly suggests the importance of the slow CHS dynamics. This result is also consistent with the slow CHS dynamics observed by Hathaway *et al.*^30^: Histone modifications around the *Oct4* locus in mouse ESCs are spatially correlated across many nucleosomes and their collective dynamics has the timescale of 
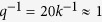
 week, which suggests 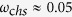, though the turnover rate of single nucleosome is as fast as 10*k*^52^. In *X*_6_(*t*) at later time instances, the tail of the population passes through the intermediate and further approaches the iPSC state. In this way, even when the average of the population represented by *X*(*t*) reverses, the tail of the population reaches the iPSC state. This behavior is consistent with the experimentally observed low efficiency of reprogramming, in which only the small portion of the cell population reaches the iPSC state.

**Figure 6:**
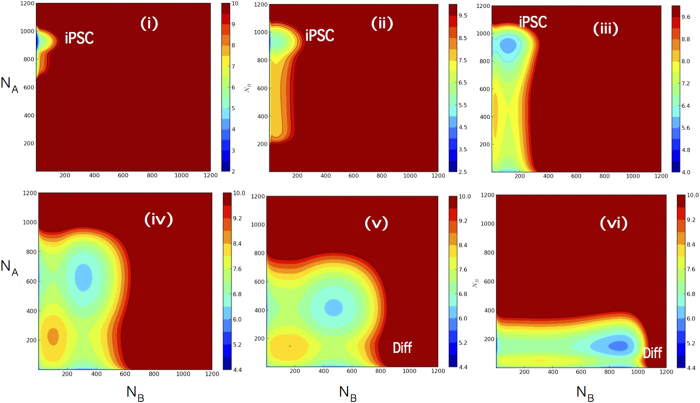
Evolution of the probability distribution *P*(*N*_*A*_,*N*_*B*_,*t*) from the iPSC to differentiated basins. Asymmetric model was simulated under the non-adiabatic condition (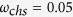) with *C*_0_ = 0. *P*(*N*_*A*_,*N*_*B*_,*t*) is shown at different time instances, (i) *t* = 0.3/*k*, (ii) 1.5/*k*, (iii) 6/*k*, (iv) 30/*k*, (v) 36/*k*, and (v) 46/*k*. At *t* = 0, the entire distribution is confined to the iPSC basin. Note the process of differentiation is independent of *γ*.

For differentiation, we use the asymmetric model because Lif is withdrawn form cultivating medium. The simulated temporal evolution of 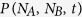 is shown in Fig. 6 with the non-adiabatic CHS dynamics (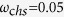). Starting from the pluripotent state by keeping *C*_0_ = 0, the population shifts along the diagonal pathway due to the bias in the asymmetric landscape, which passes through the saddle to reach the differentiated state. Though a minor population of simulated differentiating cells proceed along the epigenetic valley, major part of cells go through the diagonal pathway. We can expect that cells behave in a collective way through cell-cell communication, which should further enhance the major pathway. Thus, the difference between differentiation and reprogramming pathways is evident with the non-adiabatic CHS dynamics. In reprogramming, two genes were set to be symmetric in the binding affinity of transcription factors to gene loci in the model, but in differentiation two genes are asymmetric, which brings about the difference in pathway between reprogramming and differentiation, but this difference is realized only in the slow non-adiabatic histone switching regime.

**Figure 7:**
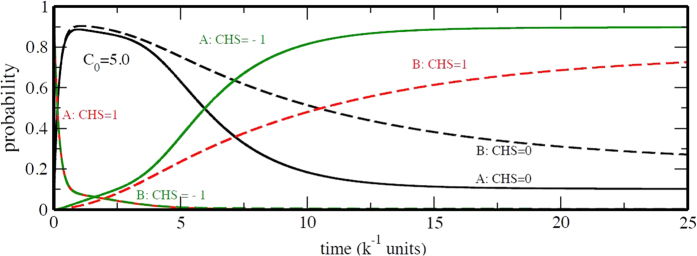
CHS evolution during reprogramming. Probability evolution of CHS for A (bold) and B (dashed) is plotted as functions of time. The intermediate is reached around *t* = 3/*k* (see Fig. 5). Symmetric model with C_0_ = 5.0*k* was used.

In Fig. 7, temporal evolution of the probability of each CHS, *P*(*s*_*α*_, *t*) for *α* = A and B, is plotted for the reprogramming process with the non-adiabatic CHS dynamics for *X*_4_(*t*). We can see that starting from the state in which *S*_*A*_ = 1 and *S*_*B*_ = −1 dominate, the system passes through the intermediate state where both 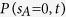 and 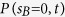 show peaks, and reaches the pluripotent state where 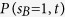 is large. In this way, the model predicts that the intermediate state in reprogramming is clearly characterised by its histone modification pattern of *s*_*A*_ = 0 and *s*_*B*_ = 0. This evolution of CHS should be examined by the single-cell observation of histone-state change.

### Kinetics of reprogramming

We investigate the role of non-adiabatic dynamics in reprogramming kinetics by simulating latencies observed in the ensemble-level experiments of Hanna *et. al.*^42^. In the report of Hanna *et al.*^42^, *N*_*col*_ founder cells infected with YF were placed in *N*_*col*_ wells on a plate at *t* = 0 to multiply and form clones. Population of these cells in each well exponentially increased from 1 to 10^6^ and saturated in *t* > 10 days. iPSC were detected through the Nanog expression measurement. The probability *Q*(*t*) that a daughter iPSC is generated from a founder cell was estimated from the observed number, *N*_nanog_^+^ (*t*), of colonies that contained Nanog expressing cells at time *t* as 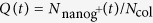.

Here, *Q*(*t*) is calculated from the simulated 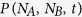 of the master equation. Let *N*_*eff*_ be the effective population size of a colony (see Methods) and *N*_*thr*_ ≈ 1, the minimum threshold number of cells to label a well as “iPSC detected”. If *R*(*t*) is the cumulative fraction of iPSC in this ensemble of cells, then *R*(*t*) = 1−*P*(*t*), where the survival probability 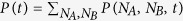 is obtained by solving the master equation and imposing an absorbing boundary condition at the iPSC state (see Methods). Assuming the cells in the effective population can be regarded as independent, we have:


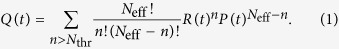


**Figure 8:**
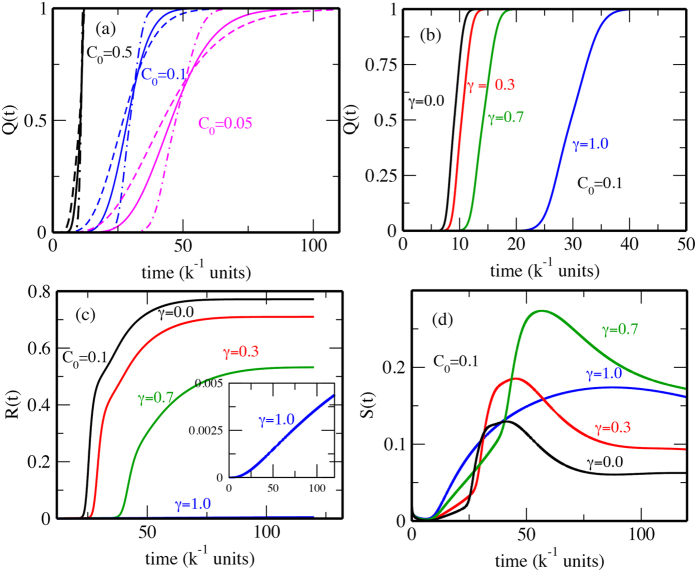
Non-adiabatic ensemble-level kinetics of reprogramming. (**a**) 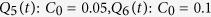 and 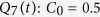 calculated with the same parameter sets as for 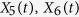 and 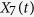 of Fig. 4b but with the absorbing boundary condition at the iPSC state with *N*_*eff*_ = 5000 (dashed),10000 (bold) and 50000 (dot-dashed). *Q*_5_(*t*) is consistent with experiments. (**b**) *Q*(*t*), (c) *R*(*t*), and (d) *S*(*t*) with various *γ* (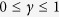) and *C*_0_ = 0.1*k*. *N*_*thr*_/*N*_*eff*_ = 5 × 10^−4^ in panel a and *N*_*eff*_ = 10000 in b. 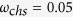 and *τ* = 100*k*^−1^ in all panels.

Figure 8a shows *Q*_5_(*t*), *Q*_6_(*t*) and *Q*_7_(*t*), which are the *Q*(*t*)s corresponding to the non-adiabatic trajectories *X*_5_(*t*), *X*_6_(*t*) and *X*_7_(*t*) of Fig. 4b. In Fig. 4b, *X*_5_(*t*) and *X*_6_(*t*) did not reach the intermediate state and revert in the epigenetic valley, which implies that the mean of 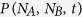 does not surpass the intermediate state with small *C*_0_. With this parameter set, however, the tail of 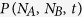 passes through the intermediate basin (Fig 5 2(i–vi)) and reaches the iPSC state, and thus brings about the slow rise of *Q*_5_(*t*) to *Q*_5_(*t*) ≈ 1 as shown in Fig. 8a. For both *Q*_5_(*t*) and *Q*_6_(*t*), *Q*(*t*) ≈ 0 initially and starts to rise at *t*_0_ and reaches *Q*(*t*) ≈ 1 at *t*_1_ showing that colonies have heterogeneous latencies. For *Q*_5_(*t*), we have *t*_0_ ≈ 28*k*^−1^ and *t*_1_ ≈ 95*k*^−1^, which agrees with the experimental values^42^: *t*_0_ ≈ 30*k*^−1^ and *t*_1_ ≈ 100–200*k*^−1^.

We find that there are two ways to accelerate reprogramming. One is to increase the efficiency *C*_0_: With the larger *C*_0_ as in *Q*_7_(*t*) (Fig. 8a), we have the sharper increase of *Q*(*t*). The other is to decrease *γ*. With *γ* = 0, *Q*(*t*) increases rapidly with *t*_0_ ≈ 8*k*^−1^ and *t*_1_ ≈ 10*k*^−1^ for *N*_*eff*_ = 10^5^, which is similar to the observed data with *t*_0_ ≈ 8*k*^−1^ and *t*_1_ ≈ 12*k*^−1^ obtained for cells in which Mbd3, a factor which binds to the methylated DNA, is silenced^43^. Thus, when YF work as histone-mark erasers with mild efficiency, reprogramming has heterogeneous latency distribution, but when YF work with high efficiency or they work as activators of pluripotency genes, reprogramming is accelerated with lesser degree of heterogeneity or is more “deterministic”^42^,^43^ in latencies.

The experimental data of Hanna *et al.* on kinetics of reprogramming^42^ was simulated also by Morris *et al.*^53^ by regarding reprogramming as a diffusion process on a phenomenological model landscape having two basins at the differentiated and iPSC states. However, role of *N*_*eff*_ was neglected in their argument^53^. In Fig. 8a, we find the slope of *Q*(*t*), which is the degree of “determinism” of reprogramming experiments^43^,^54^ to be very sensitive to *N*_*eff*_. Therefore, attention is needed to compare the data with different *N*_*eff*_.

The *γ* dependence of variation in heterogeneity of latencies is apparent in *R*(*t*) shown in Fig. 8c. Increase in *R*(*t*) is much sharper for low *γ*. The *γ* dependence of the localization properties in the *N*_*A*_-*N*_*B*_ space can be studied via the participation ratio: 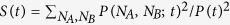, which is large when the distribution is localized and small when delocalized. Fig. 8d shows that the distribution gets more localized with increasing *γ* during the reprogramming. For *γ* = 1 population gets accumulated around the intermediate state. Localization pattern is found to be more complex in the *γ* = 0 case. These features should be verifiable by single-cell level tracking.

## Discussion

We have introduced a minimal model of reprogramming by integrating epigenetic modification dynamics with the gene expression mechanism, which provides a consistent view of pathways and kinetics. We showed how pathways are created on the epigenetic landscape aided by the slow epigenetic dynamics of collective histone-state modification. With the slow non-adiabatic epigenetic dynamics, landscape has epigenetic valleys which connect the differentiated and pluripotent cell states through the intermediate state. This pathway is consistent with the observed late activation of the pluripotency genes in the reprogramming process. The time course of the histone-state change and the extent of localization of distribution of cell states were simulated, which provide clues to examine the mechanism of the observed reprogramming process.

Based on the simulated temporal evolution of cell distribution, kinetics of reprogramming was calculated. The calculated kinetics is consistent with the experimental observation when YF is assumed to work as histone-mark erasers. With the non-adiabatic epigenetic dynamics, kinetics is sensitive to the precise mechanism of how YF work: When YF work more actively to promote expression of pluripotency genes, then the model suggests the accelerated kinetics with which reprogramming becomes “deterministic”.

We show that when we explicitly take epigenetic dynamics into account, we can explain features of the trajectory and barriers helping experimentalists with microscopic information which is otherwise difficult to obtain. The MRSA network model coupled to CHS used here is a minimal model developed for elucidating the roles of epigenetic dynamics and it is important to apply concepts and methods developed here to more realistic networks which represent antagonistic interactions between pluripotency genes and differentiation marker genes^6^,^7^,^8^,^9^,^16^.

## Methods

We first summarize in the subsection **Stochastic equations** how the gene-state transitions are modelled in the CHS coupled MRSA network we discussed. The details of the model parameterization are also discussed in this subsection. The master equation governing the stochastic equations is explained in the subsection **Master equation**. We use the proteomic field approximation^11^,^34^,^55^ to reduce the dimensionality of the master equation so that the solution is tractable computationally. Details used for kinetic calculation are explained in the subsection **Effective number of cells**.

### Stochastic equations

Reactions governing TF binding/unbinding for gene *α* = A and B are:


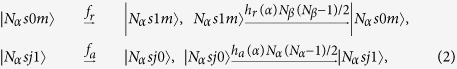


with 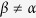. Reactions governing protein synthesis and degradation are:


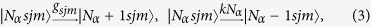


and the reactions governing CHS transitions are:


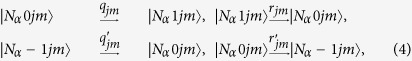


Here we use *q*_11_ = 10*q*, *q*_10_ = *q*_01_ = *q*, and *q*_100_ = 0.2*q*, while 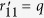, 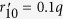, 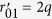, and 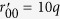. 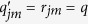 for *j* = 0, 1 and *m* = 0, 1.

### Master equation

Using the convention for indices as in *g*_*sjm*_, the master equation governing the probability distribution


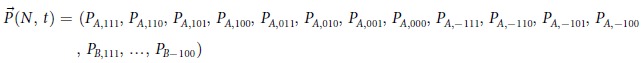



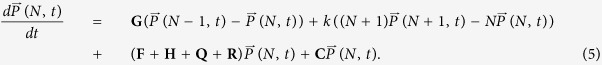


Protein generation matrix **G** is diagonal with elements 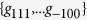, and the term with scalar 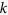 represents degradation. **F** and **H** represent unbinding and binding of TF from/to genes. 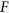 and 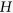 are 24-dimensional block diagonal matrices with diagonal elements 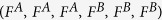 and 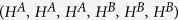;


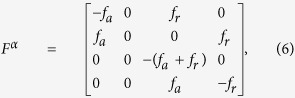



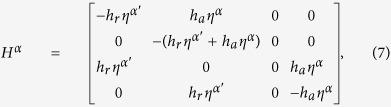


with *α* = A or B, and 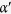 being the complement of *α*. Here 
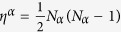
. **Q** and **R** are the CHS transition matrices defined as (with index 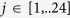):


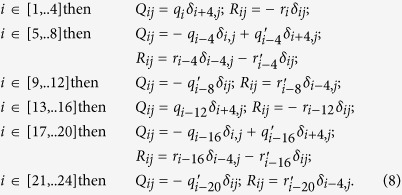


The matrix **C** represents the effects of YF. The Matrix *C* is defined through its elements: 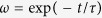. For 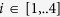, we have 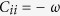, 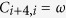, 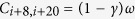, 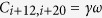, and 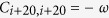. All other elements are 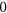. 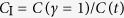 and 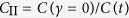.

To solve the master equation, we use the proteomic field approximation^11^,^34^,^55^, which is the the Hartree-like approximation;


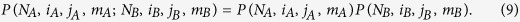


For the binding terms in **H** used in the master equation, we use an approximate form: 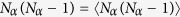. This is a reasonable approximation for copy number ≳ 10^2^. We solved the resulting proteomic field equations using the fourth order Runge-Kutta method without using the adiabatic approximation.

The trajectories 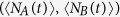 are calculated without any absorption condition. For calculating *R*(*t*) and related quantities, an absorption condition was imposed as follows: *P*(*N*_*A*_, *N*_*B*_, *t*) = 0 for *N*_*B*_ > 910, and *P*(*N*_*A*_, *N*_*B*_, *t*) was multiplied by a factor 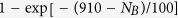 for 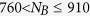.

### Effective number of cells (*N*_*eff*_)

Histone states are inherited from mother to daughter cells, there is bound to be correlation among multiple cells in a colony. The effective number of cells, *N*_*eff*_, therefore, should be smaller than the actual number of cells in a colony. We here used *N*_*eff*_ = 10^4^.

## Additional Information

**How to cite this article**: Ashwin, S. S. and Sasai, M. Effects of Collective Histone State Dynamics on Epigenetic Landscape and Kinetics of Cell Reprogramming. *Sci. Rep.* **5**, 16746; doi: 10.1038/srep16746 (2015).

## Figures and Tables

**Figure 1:**
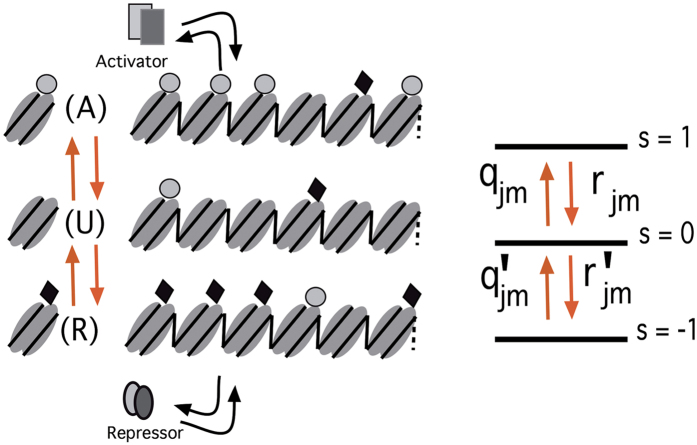
In the present model, histones are considered to be either actively marked (A: acetylated as H3K29ac and methylated as H3K4me3), repressively marked (R: deacetylated and methylated as H3K9me3) or unmarked (U; no modification or bivalently modified). The grey circles on the nucleosomes represent active histones and black diamonds represent repressive histones. Nucleosomes tend to effect neighboring nucleosomes to modify them similarly, which allows us to define collective coarse-grained histone state (CHS). When histones are collectively active, we denote CHS as *s* = 1, collectively repressive as *s* = −1, and collectively undefinable to be A or R as *s* = 0. The transition between these states depend on the repressor-binding (*j*) and activator-binding (*m*) states (subscripts of the rates *q* and *r*).
